# Effects of Let-7c on the processing of hepatitis B virus associated liver diseases

**DOI:** 10.1186/s13027-022-00458-8

**Published:** 2022-09-03

**Authors:** Like Zhang, Xia Jiang, Guiqi Wang, Tatsuo Kanda, Osamu Yokosuka, Congjie Zhai, Lei Zhang, Peng Liu, Zengren Zhao, Zhongxin Li

**Affiliations:** 1 Department of General Surgery, Hebei Key Laboratory of Colorectal Cancer Precision Diagnosis and Treatment, The First Hospital of Hebei Medical University, No.89 Donggang Road, Shijiazhuang, 050031 Hebei China; 2 Department of Gastroenterology and Nephrology, Chiba University, 1-8-1 Inohana, Chuo-ku, Chiba Japan; 3 Division of Gastroenterology and Hepatology Department of Medicine, Nihon University School of Medicine, 30-1 Oyaguchi-Kamicho, Itabashi-ku, Tokyo 173-8610 Japan

**Keywords:** Let-7c, Cancer of the liver, Chronic liver cirrhosis, Chronic hepatitis, Hepatitis B virus DNA

## Abstract

**Background:**

The most common type of cancer of the digestive system is hepatocellular carcinoma. In China, many patients harbour HBV. The lin28B/Let-7c/MYC axis is associated with the occurrence of many cancers. Therefore, we aimed to illuminate the function of the lin28B/Let-7c/MYC axis in hepatocellular carcinoma. We aimed to evaluate the critical involvement of lin28B and Let-7c in the carcinogenesis of human hepatocellular carcinoma (B-HCC).

**Methods:**

Data from the GEO database were used to analyse differentially expressed genes and IRGs. A protein − protein interaction (PPI) network and Venn diagram were generated to analyse relationships. Real-time RT-PCR, Western blotting, and cell counting kit-8 assays were used to examine the association of lin28B, Let-7c, and MYC with cell proliferation.

**Results:**

A total of 2552 functionally annotated differentially expressed RNAs were analysed in HBV patients from the GSE135860 database. In addition, 46 let-7c target genes were screened in HBV patients, and the interactions were analysed through PPI network analysis. The results confirmed that Let-7c and its target genes play a key role in HBV-related diseases. Next, we discovered a gradual decrease in Let-7c expression during the progression from HBV-associated chronic hepatitis (B-CH) and HBV-associated liver cirrhosis (B-LC) to B-HCC. We found evidence for a negative association between lin28B expression and Let-7c expression. The expression of MYC was obviously upregulated when Let-7c was inhibited.

**Conclusion:**

Our results highlight that Let-7c and lin28B participate in the carcinogenesis of HBV-associated diseases through the lin28B/Let-7c/MYC axis.

**Supplementary Information:**

The online version contains supplementary material available at 10.1186/s13027-022-00458-8.

## Background

The most common type of cancer of the digestive system is hepatocellular carcinoma (HCC). According to an investigation in 2011, the rates of malignant tumour morbidity and death have increased over time [1–3]. HBV infection may induce chronic hepatitis (HBV-CH), HBV-associated liver cirrhosis (B-LC), and hepatocellular carcinoma (HBV-HCC). In China, 80% of HCC cases are associated with HBV [4]. Additionally, in Hebei Province, HBV-HCC presents crucial risks that shorten life expectancy [5]. Ultimately, it is fundamentally important to reveal the formation mechanisms of HBV-HCC.

Various cancers, such as gastric cancers and renal cell carcinoma, have been extensively studied. The Let-7c family, MYC and lin28B are closely related in the carcinogenesis of gastric cancers and renal cell carcinoma [6–8]. Let-7 family microRNAs have biological functions and biogenesis that can be suppressed through the binding of lin28B to the terminal loop of Let-7 precursors. In the context of carcinogenesis, cell proliferation, apoptosis and migration, this process significantly decreases Let-7 target gene expression [9, 10].

A homologous gene of lin28A called lin28B contains a retroviral-type zinc finger and a cold shock domain [11]. The ubiquitous expression of lin28A and lin28B in embryonic stem cells is associated with maintenance of pluripotency and embryonic differentiation [12]. The significant prognostic value of lin28A and lin28B has been confirmed [13]. However, lin28B has demonstrated more frequent upregulation and a close relationship with various human cancers [14, 15]. Additionally, lin28B promotes cancer cell migration and cancer progression, metastasis, and recurrence among colorectal cancer patients.

In recent studies, Let-7 family members have been associated with the tumour microenvironment and clinical outcome [16, 17]. In this study, we assessed the expression of all Let-7 family members in serum specimens while analysing the significance of the correlations between Let-7c, lin28B, and MYC. As Let-7c inhibits replication of hepatitis C virus, we also focused on the physiological action on HCC tissues [18]. In HCC tissues, we measured the expression of Let-7 family members and investigated whether Let-7c expression correlates with HCC TNM stage. Additionally, Let-7c is known to have a close link with HCC prognosis [19, 20]. Lin28B and Let-7c also participate in other kinds of cancers. In papillary thyroid carcinoma, a study showed that the Lin28A/Let-7a/MYC pathway plays an important role in cell growth and malignant behaviour [21]. Additionally, in alcoholic liver injury, there is negative feedback between lin28B and Let-7 in the activation of hepatic stellate cells [22]. Therefore, we tested the expression of all mature Let-7 family members and MYC in serum samples taken from patients carrying HBV. From these data, we analysed the overall effects of the lin28B/Let-7c/MYC axis on hepatic carcinogenesis.

## Methods

### Data collection

The dataset selection criteria were as follows: 1. transcript profiling (transcriptomic) data including RNA-seq data; 2. accessible basic clinicopathological parameters (stage and OS information included); and 3. sample size exceeding 50 subjects. 1 dataset, namely, GSE135860, was extracted from https://www.ncbi.nlm.nih.gov/geo/query/acc.cgi?acc=GSE135860. We assessed the expression profiles of each dataset manually. A total of 6 datasets were enrolled. The GEO approved publication guidelines were complied with, and the data were extracted from the database. Therefore, the approval of the ethics committee was not required.

### Identification of DEGs

We utilized R software’s limma package [23] to conduct differential gene analysis by using cut-off values set at false discovery rate (FDR) < 0.05 and log2 | fold change |> 1. Based on these results, we acquired an itemized list of significant DEGs (differentially expressed genes) in the expression matrix. R software’s limma package was used to carry out the differential gene analysis. A false discovery rate (FDR) < 0.05 and log2 | fold change |> 1 were employed as cut-off values.

### IRG function and pathway enrichment analyses

Necessary information for IRG biological pathway and functional analyses was obtained. A GO analysis of biological process (BP), molecular function (MF), and cellular component (CC) terms was performed. The R package clusterProfiler [24] was used for Kyoto Encyclopedia of Genes and Genomes (KEGG) pathway enrichment analysis. A false discovery rate (FDR) < 0.05 was considered statistically significant.

### Protein–protein interaction (PPI) network

The PPI network was generated by the STRING database [25] through Cytoscape software [26]. Individual networks with 10 or more nodes were included, and those with fewer than 10 nodes were excluded. In each network node, the grade of the connectivity was computed, and the clusters were collected according to their typology to trace densely connected regions by molecular complex detection (MCODE).

### Clinical samples

All serum samples, which included the serum samples of eighty-nine HBV-CH, ten HBV-LC, and eight HBV-HCC patients, were collected from Chiba University Hospital in Japan. The diagnosis of all the patients was based on pathological sections taken during surgical resection or liver biopsy, along with the detailed data of the B-HCC and B-LC/CH patients, which is itemized in Table 1. Use the METAVIR Score to understand the stages of liver fibrosis, and patients with F4 grade was diagnosed LC. The clinical characteristics of HBV markers were showed in Table 2. Serum samples and tissue samples were stored at − 20 °C and − 80 °C freezers, respectively, until need for use arose.

**Table 1:** Clinicopathological characteristics of B-CH, B-LC and B-HCC patients

Groups	Observation index	P50 (P25, P75)
B-CH	n	90
	Age	38.00 (33.00, 47.00)
	Gender	
	Female	23
	Male	67
	AST (IU/L)	58.00 (32.25, 104.50)
	ALT (IU/L)	93.50 (43.00, 177.75)
	γGTP (IU/L)	39.00 (26.00, 64.50)
	PLT (× 10^9^/L)	178.00 (150.25, 210.25)
	ALB (g/L)	4.20 (4.03, 4.48)
	HBV-DNA	7.10 (4.20, 7.60)
B-LC	n	10
	Age	53.50 (48.75, 54.75)
	Gender	
	Female	3
	Male	7
	AST (IU/L)	69.00 (45.25, 82.00)
	ALT (IU/L)	83.00 (40.75, 100.00)
	γGTP (IU/L)	48.50 (39.00, 78.25)
	PLT (× 10^9^/L)	138.00 (87.00, 180.25)
	ALB (g/L)	4.00 (3.28, 4.08)
	HBV-DNA	6.20 (3.50, 7.30)
B-HCC	n	8
	Age	50.00 (44.00, 65.00)
	Gender	
	Female	4
	Male	4
	AST (IU/L)	33.50 (25.50, 42.75)
	ALT (IU/L)	35.00 (21.75, 62.00)
	γGTP (IU/L)	42.50 (22.00, 71.50)
	PLT (× 10^9^/L)	109.50 (86.50, 138.25)
	ALB (g/L)	3.95 (3.80, 4.13)
	HBV-DNA	N. D

**Table 2:** HBV serological markers characteristics of participants, number (%)

		HBsAg	anti-HBs	HBeAg	anti-HBe	DEP^a^	HBV DNA
Total	+	100 (92.59)	3 (2.78)	74 (68.52)	45 (41.67)	13 (12.04)	89 (82.41)
	−	8 (7.41)	105 (97.22)	34 (31.48)	63 (58.33)	95 (87.96)	19 (17.59)
B-CH	+	82 (91.11)	3 (3.33)	68 (75.56)	31 (34.44)	11 (12.22)	79 (87.78)
	−	8 (8.89)	87 (96.67)	22 (24.44)	59 (65.56)	79 (87.78)	11 (12.22)
B-LC	+	10 (100.00)	0	5 (50.00)	7 (70.00)^c^	2 (20.00)	9 (90.00)
	−	0	10 (100.00)	5 (50.00)	3 (30.00)	8 (80.00)	1 (10.00)
B-HCC	+	8 (100.00)	0	1 (12.50)^d^	7 (87.50)^d^	0	1 (12.50)^d,e^
	−	0	8 (100.00)	7 (87.50)	1 (12.50)	8 (100.00)	7 (87.50)
*P* value^b^		*N.S*	*N.S*	< 0.05	< 0.05	*N.S*	< 0.05

^a^DEP: dual-positivity for both HBeAg and anti-HBe

^b^Compared 3 groups (B-CH, B-LC and B-HCC), calculated by Chi-square test

^c^*P* < 0.05, compared between B-CH and B-LC, calculated by Chi-square test

^d^*P* < 0.05, compared between B-CH and B-HCC, calculated by Chi-square test

^e^*P* < 0.05, compared between B-LC and B-HCC, calculated by Chi-square test

### Cell culture

HepG2.2.15 cells containing the complete HBV genome and supporting the assembly and secretion HBV DNA, were obtained from translational medicine research center (North Sichuan Medical College, Nanchong, China). We cultured human hepatoma HepG2 cells in Dulbecco’s modified Eagle’s medium, which we obtained from Invitrogen (Carlsbad, CA, USA). More specifically, the medium contained 10% heat-inactivated foetal bovine serum, 100 units/ml penicillin, and 100 µg/ml streptomycin from Sigma (St. Louis, MO, USA), and cells were cultured under a 5% CO_2_ atmosphere at 37 °C.

### Real-time reverse transcription quantitative polymerase chain reaction (Real-time RT–qPCR)

We extracted total RNA from serum samples and HBV-paired serum samples. We used the standard protocols from the TaqMan microRNA Reverse Transcription kit (Applied Biosystems, California, USA) and TaqMan Universal Master Mix (Applied Biosystems) to perform reverse transcription and real-time PCR, respectively. The lin28B primers were as follows: 5’-CATGGTGGCAAACTGCCCACATAA-3’ (forwards) and 5’-TTCGTGGAGGAAGCTTCTTGAGGT-3’ (reverse). To normalize variance, we utilized GAPDH as an endogenous control. The primers were 5’-AGCCTCAAGATCATCAGCAATG-3’ (forwards) and 5’-TGTGGTCATGAGTCCTTCCACG-3’ (reverse). We obtained the Let-7c primers (479,365, Applied Biosystems, California, USA) from Applied Biosystems, and cel-miR-39 and U6 (4,427,975, Applied Biosystems, California, USA) were used as endogenous controls. We utilized relative quantification (2^−ΔCT^) to compute fold changes.

### Cell proliferation assay

Using a cell counting kit-8 (CCK-8) assay kit (Dojindo, Kyushu Island, Japan), we assessed cell proliferation. In 96-well plates, we cultured HepG2 cells separately at a density of 5000 cells/well overnight. We transfected the cultures with Let-7c inhibitor and control. After 24 h, 48 h, 72 h, and 96 h of transfection, 10 μl of CCK8 solution was added to each well. The cells were cultured for 3 h. Using a Glomax multidetection system (Promega, Wisconsin, USA) according to the manufacturer’s instructions, we detected the absorbance level at 450 nm.

### Western blotting

The cells were transfected with a Let-7c inhibitor. Additionally, 1X SDS lysis buffer was used to lyse the cells. SDS-PAGE was used to isolate proteins, which were transferred onto PVDF membranes. MYC (#9402, Cell Signaling Technology, Massachusetts, USA) and GAPDH (10,494–1-AP, Proteintech, Wuhan, Hubei, China) staining was detected. The Odyssey CLx Infrared Imaging System (LI-COR Biosciences, Lincoln Nebraska, USA) was used to visualize immunoreactive bands.

### Statistical analysis

We executed the data analysis using SPSS Graduate Pack 21.0 (IBM, New York, USA), GraphPad Prism 5 software (GraphPad Software, San Diego, USA), or Student’s t test. The cut-off for statistical significance was *P* < 0.05.

## Results

### Differentially expressed RNAs and functional annotation in HBV patients from the GEO database

**Fig. 1:**
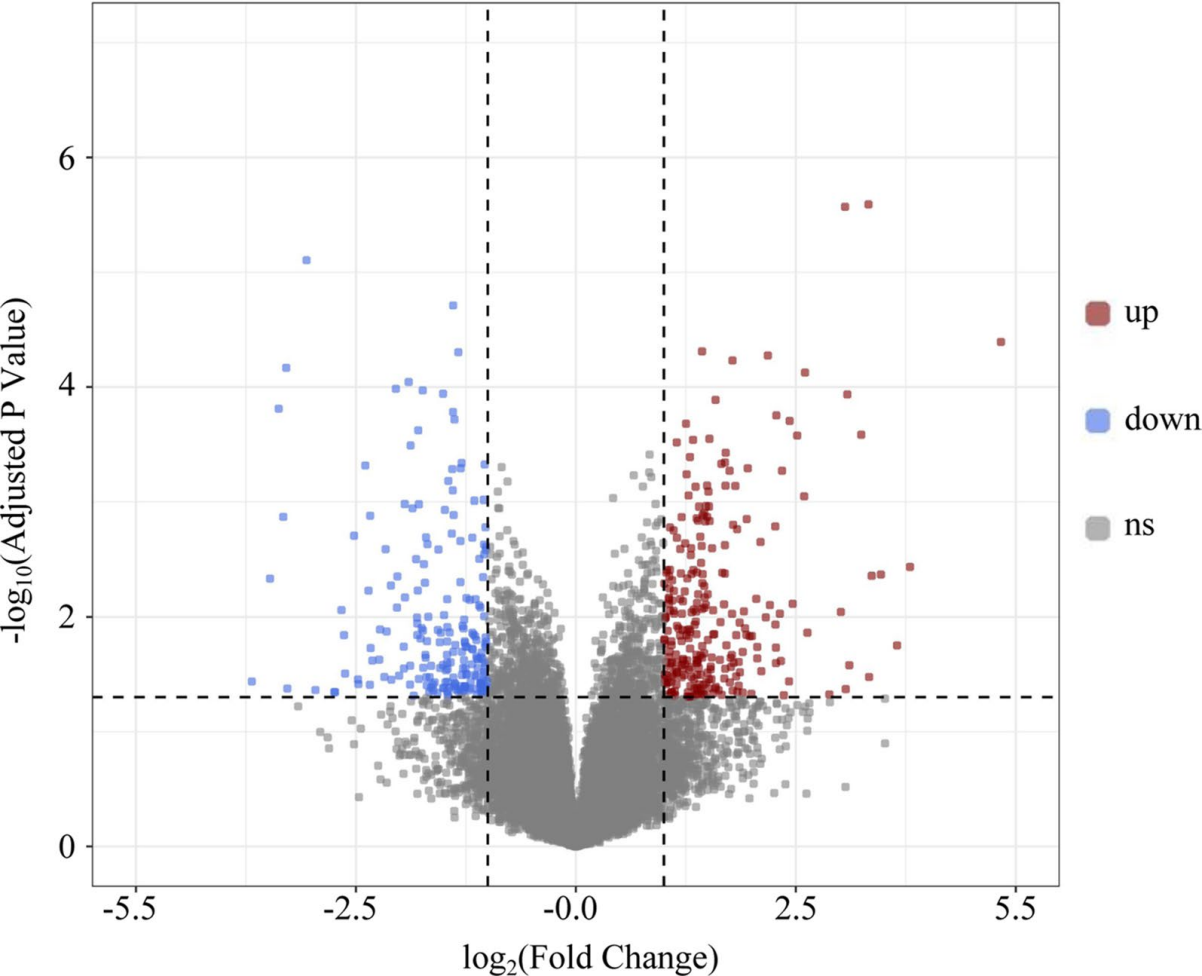
Volcano plot of differentially expressed mRNAs

**Fig. 2:**
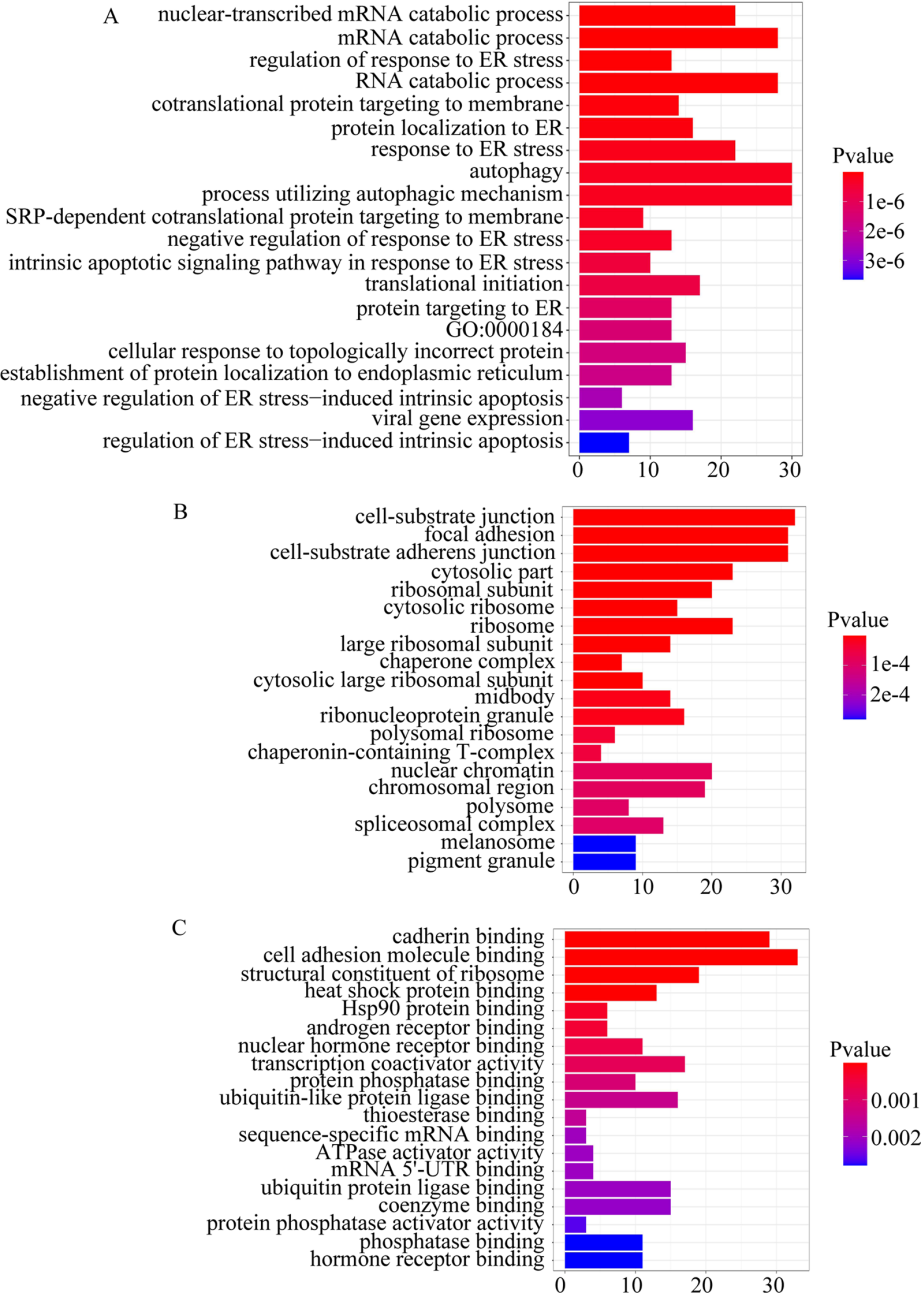
Gene ontology and KEGG pathway functional enrichment analyses of the differentially expressed mRNAs **a** GO biological function terms **b** GO cell component terms **c** GO molecular function terms

**Fig. 3:**
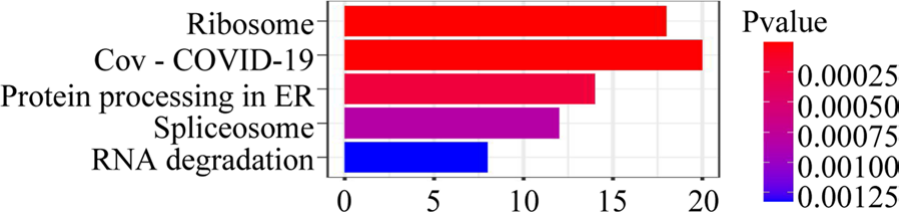
Gene ontology and KEGG pathway functional enrichment analyses of the differentially expressed mRNAs. KEGG pathway functional classification and annotation

A total of 3 HBV samples and 3 control samples were obtained from the GSE135860 dataset, with 23,949 RNAs measured for each. According to the differential analysis by the Wilcoxon test, we identified 2552 mRNAs as significantly differentially expressed in the HBV compared with normal tissue samples, and the results are displayed in the volcano plot in Fig. 1. We also analysed these mRNAs using the R software package clusterprofifiler to identify the functions linked to the different mRNAs. This evaluation revealed enrichment of 321 GO terms along with 9 KEGG pathways (FDR < 0.05). We chose to show the top 9 GO terms and 20 KEGG pathways of the DEmRNAs based on the gene count in Figs. 2 and 3.

### The relationships among the let-7c target genes expressed in HBV patients

**Fig. 4:**
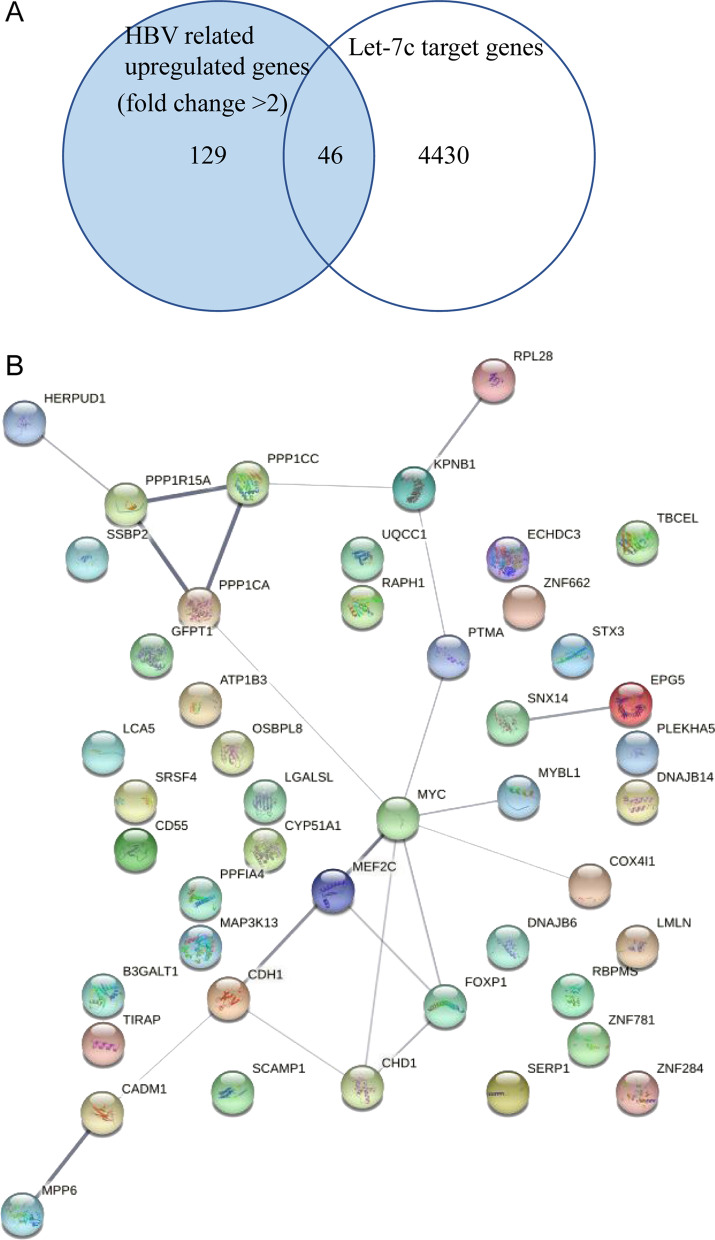
**a** Venn diagram of HBV-related upregulated genes and Let-7c target genes **b** Network map showing the interplay of Let-7c target genes

We found 129 HBV-related upregulated genes in the GSE135860 dataset, and 46 common genes were identified by the intersection of the 129 HBV-related upregulated genes with 4430 Let-7c target genes from TargetScan, Tarbase, miRDB and miRanda. Based on the resulting 46 genes, a Venn diagram was constructed (Fig. 4a). Using STRING, we generated a PPI network that included 22 edges and 46 nodes to study the interactions among the MYC genes (Fig. 4b). We found that MYC was most strongly correlated with other genes in the PPI network, including CDH1, CHD1, COX4I1, FOXP1, MEF2C, MYBL1, PPP1CA and PTMA.

### There was a significant reduction in the expression of Let-7c in HepG2.15 cells

**Fig. 5:**
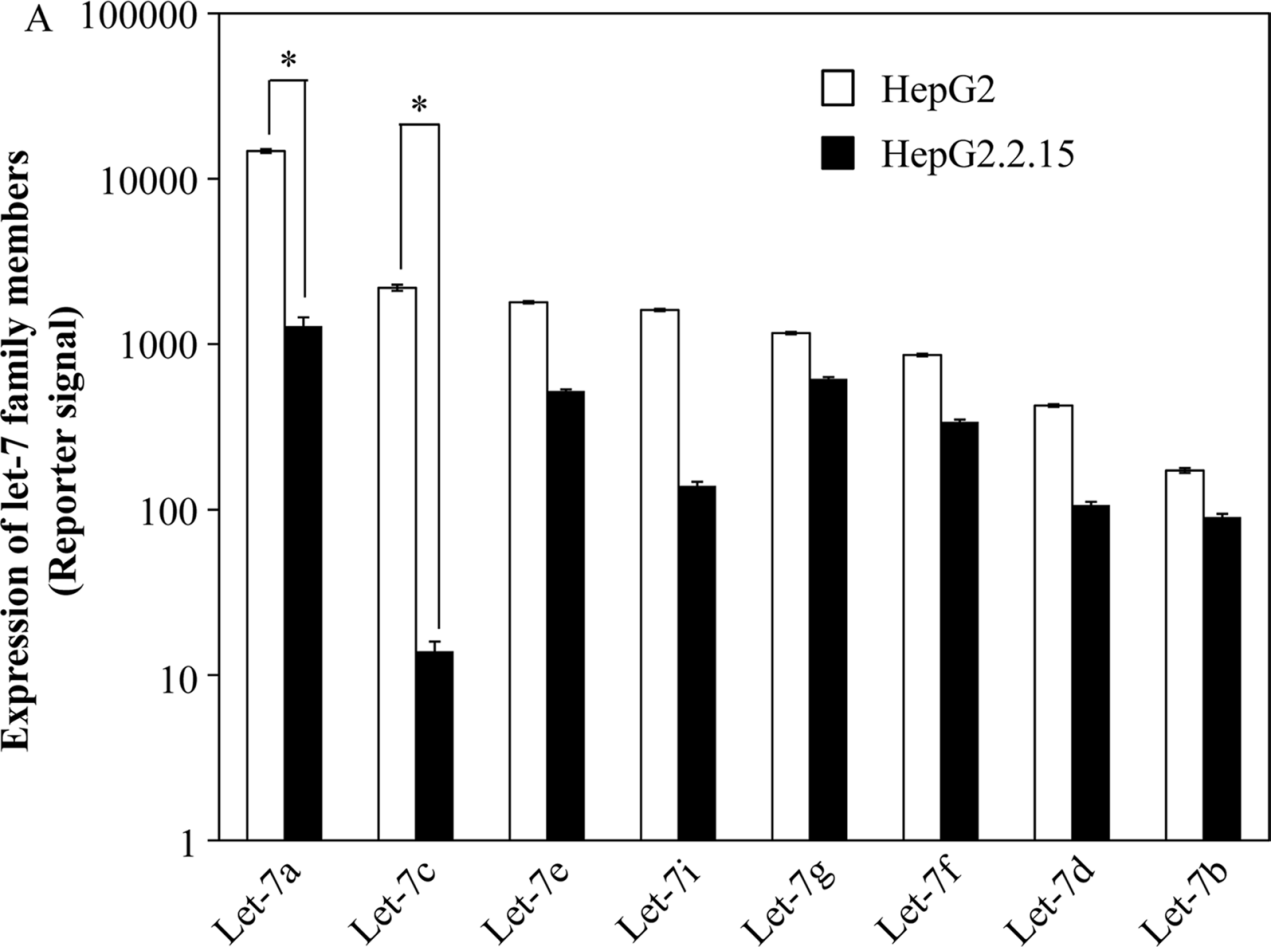
Expression of the Let-7 family. The expression of Let-7 family miRNAs was measured in hepatocytes. The HepG2.2.15 group was infected with HBV. * Means *P* < 0.05

In our study, we analysed the expression of all Let-7 family members, including Let-7a-5p (Let-7a), Let-7b-5p (Let-7b), Let-7c, Let-7d-5p (Let-7d), Let-7e-5p (Let-7e), Let-7f-5p (Let-7f), Let-7 g-5p (Let-7 g), Let-7i-5p (let-7i), Let-7a-3p, Let-7a-2-3p, Let-7b-3p, Let-7d-3p, Let-7e-3p, Let-7f-1-3p, Let-7f-2-3p, Let-7 g-3p, let-7i-3p, and miR-98-5p. Our results demonstrated that the expression of Let-7a-3p, Let-7a-2-3p, Let-7b-3p, Let-7d-3p, Let-7e-3p, Let-7f-1-3p, Let-7f-2-3p, Let-7 g-3p, let-7i-3p, and miR-98-5p was less than that in normal hepatocytes (data not shown). In hepatocytes, members with increased expression were Let-7a, Let-7c, Let-7e, Let-7i, and Let-7 g, while those with decreased expression were Let-7f, Let-7d, and Let-7b. The member with the highest expression in hepatocytes was Let-7a. In HBV-associated hepatic tumour tissues (compared to adjacent tissues), the downregulation of almost all Let-7 family members expression was evident along with significant reduction of Let-7c expression (*P* < 0.05) (Fig. 5). These results led us to focus on Let-7c for further research.

### Let-7c inhibition promoted HepG2 cell proliferation and the expression of MYC

Shi demonstrated a downward trend in the expression of Let-7c from normal control to chronic hepatitis, liver cirrhosis, adjacent nontumour, and HCC samples [20]. However, the result was mainly derived from liver tissues. To date, the expression of Let-7c in serum samples has not been studied. Here, the expression of lin28B in B-HCC was elevated. The data showed that lin28B and Let-7c had a negative correlation, which led to the analysis of Let-7c expression in B-HCC.

**Fig. 6:**
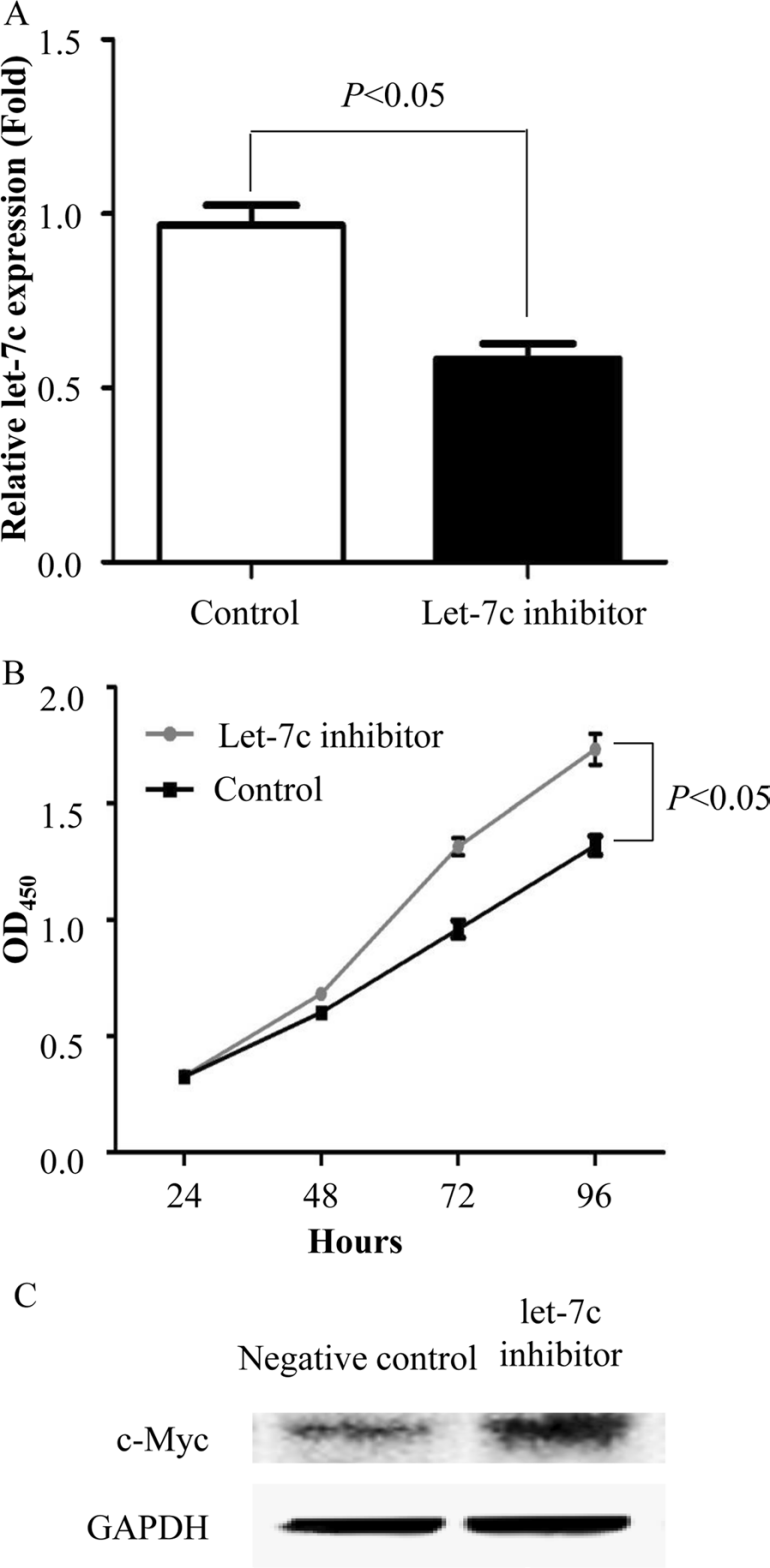
Cell proliferation and MYC expression were increased after Let-7c was inhibited. **a** The expression of Let-7c was obviously inhibited by the Let-7c inhibitor. **b** Cell proliferation was promoted after Let-7c was inhibited. The HepG2 cell line was used, and cell proliferation was detected by CCK-8 assay. **c** Expression of MYC was examined by Western blotting. All the experiments were independently repeated three times

We inhibited Let-7c in HepG2 cells. The results showed successful significant inhibition of Let-7c expression (*P* < 0.05) (Fig. 6a). We also used the CCK-8 assay to examine the proliferation of HepG2 cells. The results showed that proliferation was evidently promoted after 24 h of cultivation. However, after 72 h, the rate of promotion was slightly decreased (Fig. 6b).

After Let-7c was inhibited, we used the CCK-8 assay to detect the proliferation of HepG2 cells. We cultured HepG2 cells for 96 h. The proliferation of the HepG2 treatment groups was promoted when the cells were cultured for more than 24 h. Additionally, the treatment groups were maintained for 48 h, 72 h, and 96 h of cultivation (Fig. 6b). Western blotting detected the expression of c-Myc, which was obviously promoted when Let-7c was inhibited (Fig. 6c). The inhibition of Let-7c expression led to the upregulation of MYC expression.

### The expression of lin28B in B-HCC samples was upregulated: the expression of Let-7c decreased gradually with liver cirrhosis development

**Fig. 7:**
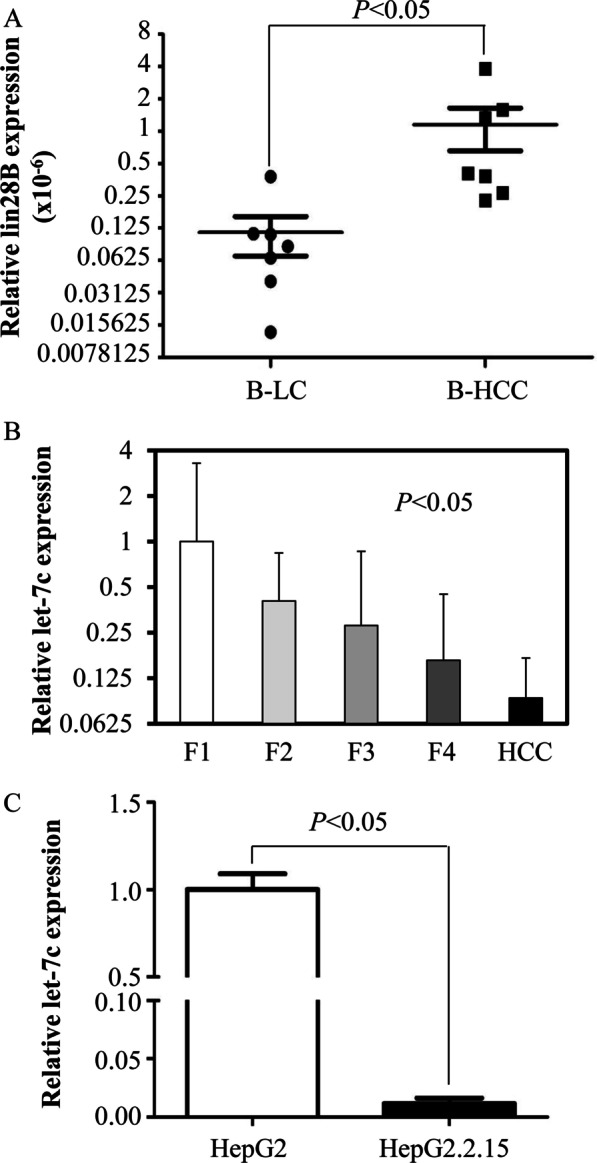
Lin28B expression and Let-7c expression in B-HCC and B-LC/CH serum samples. **a** lin28B expression in B-HCC and B-LC samples. **b** Let-7c expression in B-HCC and B-LC/CH serum samples. **c** Let-7c expression in the HepG2 group and HepG2.2.15 group. The last group was infected by HBV. All the data were measured by real-time PCR, and experiments were independently repeated three times

In contrast to the B-LC samples, there was clear upregulation of lin28B in the B-HCC samples. We used real-time PCR to detect expression. Overall, the results were statistically significant at *P* < 0.05 (Fig. 7a). Furthermore, we detected the expression of Let-7c in 99 cirrhosis samples and 8 B-HCC samples. Among all of the samples, F1 samples showed the highest expression of Let-7c, while B-HCC samples showed the lowest expression of Let-7c. This gradual decreasing trend in the expression of Let-7c with progression of liver cirrhosis is an important discovery (Fig. 7b).

In addition, the expression of Let-7c was detected when samples were divided into a HepG2 group and HepG2.2.15 group infected by HBV. The results were encouraging. The expression of Let-7c was significant altered (*P* < 0.05) (Fig. 7c).

### The expression of Let-7c was related to the levels of HBV DNA and total bilirubin (T-Bil) in serum

**Fig. 8:**
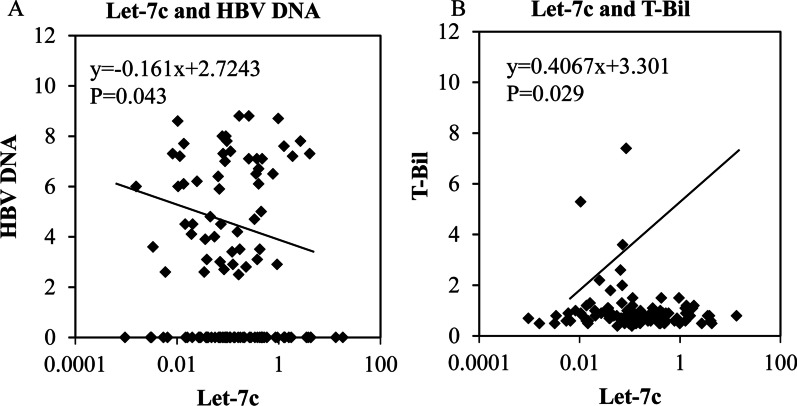
Relationships between the expression of Let-7c, HBV DNA level and T-Bil in serum samples. **a** The expression of Let-7c was negatively correlated with HBV DNA level. **b** The expression of Let-7c was positively correlated with T-Bil in serum samples

The expression of Let-7c was negatively correlated with the level of HBV DNA, and the result was statistically significant (*P* = 0.043) (Fig. 8a). The levels of HBV DNA, T-Bil and Let-7c in HBV-CH, HBV-LC and HBV-HCC were analyzed separately. All data of HBV DNA, T-Bil and Let-7c had no significant difference among three groups (Additional file 1: Fig. S1). The expression of Let-7c was positively correlated with the level of T-Bil in serum. This result was also statistically significant (*P* = 0.029) (Fig. 8b).

## Discussion

In our study, there was evident downregulation of the expression of Let-7 family members in hepatic tumour tissues, with Let-7c having the most significant reduction (*P* < 0.05) in HBV-associated hepatic tumour tissues. The expression of lin28B in B-HCC tissue samples was apparently higher than that in B-CH and B-LC samples. Additionally, Let-7c expression was negatively associated with lin28B expression and MYC expression and negatively correlated with the proliferation of hepatocytes. Overall, a gradual decrease in Let-7c expression occurred with liver cirrhosis development, and Let-7c expression was the lowest in the B-HCC samples. By analysing the clinical data of HBV patients, we found that Let-7c was related to HBV DNA level, which is related to disease severity. To our surprise, mild elevation of Let-7c was positively correlated with T-Bil. These results show that Let-7c may be used as a serum biomarker of HBV-associated progression, and the importance of Let-7c in HBV-related liver cancer was clarified.

Our results indicate that lin28B has a close connection with Let-7c. High expression of lin28B was associated with a low level of Let-7c. A high level of Let-7c was associated with low expression of lin28B. We concluded that a double-negative feedback loop between lin28B and Let-7 could explain this result [11]. Two Cys-Cys-His-Cys type zinc finger domains at the C-terminus and the cold-shock domain at the N-terminus comprise the RNA binding domains of lin28B [27]. Although the correlation between Let-7c and Lin28B expression was not analyzed in more other hepatocellular carcinoma cells in this study, there have been relevant literatures to clarify their regulatory mechanism. Lin28B can prevent precursors of Let-7 from becoming mature Let-7 by binding to Let-7 precursors via the RNA binding domains [11]. David H. also pointed out that lin28B can block the biological function of Let-7 through terminal uridylic transferase 4 (TUT4). This causes uridylation of the 3’-terminal of the Let-7 precursors and the subsequent degradation of the Dis312 exonuclease [28], which leads to the downregulation of Let-7. On the other hand, by interacting with the complementary site of lin28B, Let-7 can also inhibit the expression and function of lin28B [29]. This double negative-feedback loop affects tumorigenesis and the migration, metastasis, and treatment sensitivity of cancer cells. Blair B. Madison’s research discovered how lin28B could stimulate growth and tumorigenesis of the intestinal epithelium through Let-7c. The hypertrophy and Paneth cell depletion caused by lin28B can be reversed through the expression of Let-7c [30].

Reports suggest a close relationship of the Let-7 family with MYC. In our study, we also found that when Let-7c was inhibited, the expression of MYC was promoted, indicating that Let-7a, Let-7c, and Let-7 g could interact with the 3’-UTR of MYC, resulting in the inhibition of the expression of MYC [8]. Moreover, another study used a reporter assay to show that the luciferase activity in the wild-type group was significantly decreased by Let-7b mimic transfection. However, the Let-7 binding site-mutant MYC 3’UTR reporters did not show this pattern. This study suggests that the sequence-specific suppression of MYC by Let-7b is dependent on the binding of Let-7b to the 3’UTR of MYC [31]. The research of Valerie B. Sampson revealed that the overexpression of Let-7a downregulated the level of Myc RNA and protein. Furthermore, downregulation of Myc expression led to elevation of Let-7a [7].

The deregulation of MYC is related to many kinds of cancers, such as lung carcinoma, glioma, colon adenocarcinoma, and breast adenocarcinoma. Wang’s research highlights how depletion of MYC can inhibit the proliferation of normal human and cancer cells caused by MYC. This inhibition occurs at different phases in different cancer cell lines [32]. Overexpression of MYC can activate an impaired DNA damage response, which leads to genomic instability and tumor progression [33]. Moreover, MYC acts as an oncoprotein. However, it is difficult to target MYC [34] because not does not have enzymatic activity, making cofactors and downstream factors important for treatment. Den verified the upregulation of Aurora kinase A transcripts by MYC. Moreover, blocking the activity of Aurora kinases A leads to transient mitotic arrest, which makes it a therapeutic target to treat tumors [35].

The progression from B-LC to B-HCC has been recognized. Our results showed that Let-7c is negatively related to liver cirrhosis and HBV DNA level. Research has pointed out that a decrease in HBV DNA level is important for the development of B-HCC in liver cirrhosis patients [36]. HBV DNA copies > 4 log (10) indicates a higher risk of liver cirrhosis [37]. Therefore, Let-7c may be an essential factor for the process. However, the mechanism connecting Let-7c with the HBV DNA level requires further research.

To our surprise, Let-7c had a positive relationship with T-Bil in B-HCC. A study showed a negative correlation between a high level of serum T-Bil and cancer development [38]. In a European study and a Korean study, serum T-Bil was also negatively related to lung cancer risk [39, 40]. Another article pointed out that a mildly increased concentration of T-Bil may be related to protective effects in people with cancer [41]. Generally, the level of HBV DNA is positively related to the level of T-Bil to a large extent. In this study, there were negative correlation between Let-7c and HBV associated diseases progression/HBV DNA, but the Let-7c expression was somewhat unreasonably positively related to T-Bil. Therefore, we examined the data and patients’ clinical characteristics in this study, and found that the level of T-Bil were normal in most of the patients (96.30%, *N* = 104), while only in 4 patients T-Bil levels exceeded the clinical normal range (3.70%, *N* = 4). Although there was no significant difference in HBV DNA levels between normal T-Bil group and high T-Bil group (Additional file 2: Fig. S2). But interestingly, in high T-Bil patients, the level of HBV DNA is strongly positively related to T-Bil levels in our cohorts; while there are no relationship or has a little bit negative association between HBV DNA and T-Bil in normal T-Bil patients. We considered the reason may be that the percentage of the dual-positivity for both HBeAg and anti-HBe patients (12%) is higher than common (0.2–5.9%) [42, 43], particularly in B-LC group (20%). So, our results may only mirror the correlation between normal range T-Bil and Let-7c expression. More evidence and further study were needed to explicit the correlation.

## Conclusion

In summary, Let-7c may participate in HBV-associated carcinogenesis through the lin28B/Let-7c/MYC axis. However, further investigations and studies of these topics are needed.

## Supplementary Information

**Additional file 1**: **Fig. S1**. The levels of HBV DNA, T-Bil and Let-7c in HBV-CH, HBV-LC and HBV-HCC.

**Additional file 2**: **Fig. S2**. The levels of HBV DNA between normal T-Bil group and high T-Bil group.

## Data Availability

All data generated during this study are included in this published article. The data that support the findings of this study are available from [https://www.ncbi.nlm.nih.gov/geo/query/acc.cgi?acc=GSE135860] but restrictions apply to the availability of these data, which were used under license for the current study, and so are not publicly available.

## Abbreviations

B-HCC: Hepatitis B virus-associated hepatocellular carcinoma
B-CH: Hepatitis B virus-associated chronic hepatitis
B-LC: Hepatitis B virus-associated liver cirrhosis
HCC: Hepatocellular carcinoma
T-Bil: Total bilirubin
