# Supplementary figures and images for: Effects of Let-7c on the processing of hepatitis B virus associated liver diseases
Source: Infect Agent Cancer. 2022 Sep 3;17:46. doi: 10.1186/s13027-022-00458-8 (PMC9440497; doi:10.1186/s13027-022-00458-8)

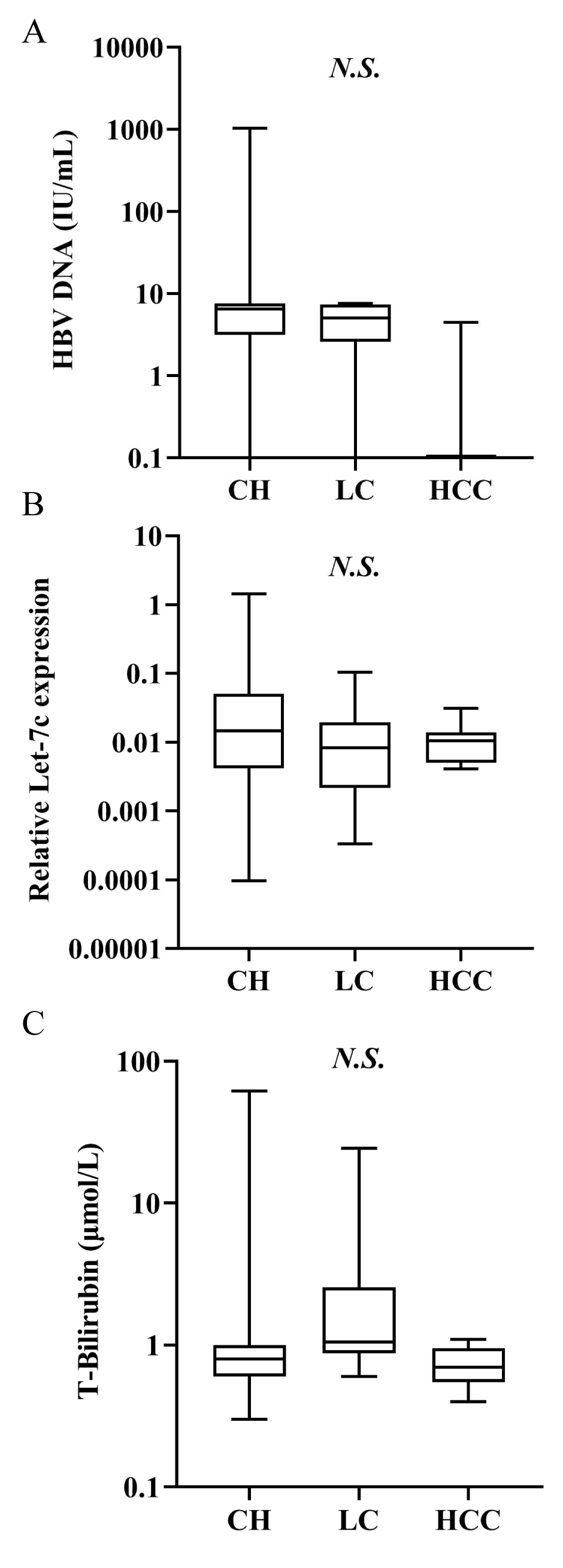

Supplement: Supplementary file 1 — Additional file 1: Fig. S1. The levels of HBV DNA, T-Bil and Let-7c in HBV-CH, HBV-LC and HBV-HCC. [file 13027_2022_458_MOESM1_ESM.tif]

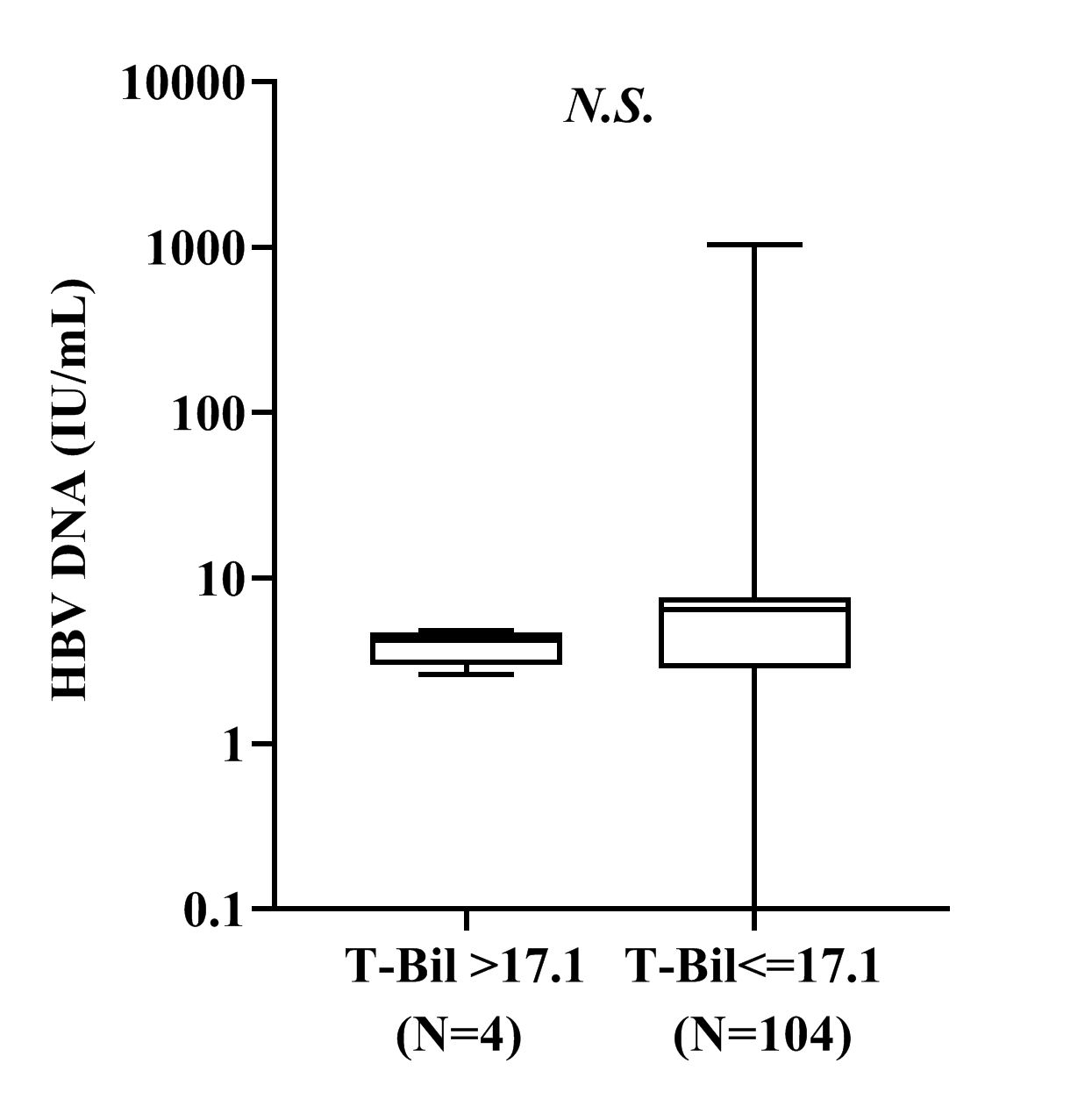

Supplement: Supplementary file 2 — Additional file 2: Fig. S2. The levels of HBV DNA between normal T-Bil group and high T-Bil group. [file 13027_2022_458_MOESM2_ESM.tif]
